# Association of adherence measured by self-reported pill count with achieved blood pressure level in hypertension patients: a cross-sectional study

**DOI:** 10.1186/s40885-022-00195-5

**Published:** 2022-04-15

**Authors:** Iin Ernawati, Eziah Ika Lubada, Ria Lusiyani, Rahmad Aji Prasetya

**Affiliations:** Department of Clinical and Community Pharmacy, Akademi Farmasi Surabaya, Surabaya, East Java Indonesia

**Keywords:** Hypertension, Blood pressure, Medication adherence, Pill count, Antihypertensive drug

## Abstract

**Background:**

Medication adherence plays an essential role in controlling blood pressure to reduce morbidity and mortality of hypertension disease. Thus, this study aimed to determine the association of medication adherence measured by self-reported pill count with blood pressure levels among patients at several community health centers in Surabaya.

**Methods:**

Adherence was assessed using the pill count method by comparing the total number of antihypertension drugs taken with the prescribed drugs. The inclusion criteria involved hypertensive patients who received antihypertension drugs, specifically adults and elderly, except the pregnant woman. The patient blood pressure was measured by healthcare workers in the targeted community health centers. Descriptive and multivariable logistic regression analyses were performed to assess factors associated with medication adherence with blood pressure levels.

**Results:**

A total of 264 hypertensive outpatients participating in this study, 77.65% of participants were adherent to antihypertensive drugs based on the pill count method, and 40.91% of participants had controlled blood pressure. Patients with uncontrolled blood pressure were about six times (adjusted odds ratio [AOR]: 6.15; 95% confidence interval [CI]: 2.694–14.039; *P* = 0.000) more likely to have non-adherent medication than patients with controlled blood pressure. Reciprocally, non-adherent participants (pill count < 80%) were about six times (AOR: 6.081; 95% CI: 2.672–13.838; *P* = 0.000) more likely to have uncontrolled blood pressure compared to adherent patients (pill count ≥ 80%). Age less than 40 years old (AOR: 5.814; 95% CI: 1.519–22.252; *P* = 0.01) and having middle school educational level (AOR: 0.387; 95% CI: 0.153–0.974; *P* = 0.045) were found to be independent factors associated with uncontrolled blood pressure.

**Conclusions:**

The result showed that non-adherence to antihypertension drugs is associated with uncontrolled blood pressure. Then, age could be associated with uncontrolled blood pressure. Thus, pharmacists and other healthcare providers should pay attention to improving medication adherence and maintaining the controlled blood pressure.

**Supplementary Information:**

The online version contains supplementary material available at 10.1186/s40885-022-00195-5.

## Background

Hypertension is a non-communicable disease that is currently increasing in prevalence. Based on a national population survey in 2018, the prevalence of hypertension in Indonesia was 34.1% (age ≥18 years old) [1]. Furthermore, data from the World Health Organization (WHO) showed that around 1.13 billion people had hypertension in 2015, meaning that one out of three people worldwide was potentially diagnosed with hypertension. The hypertension case continues to increase and will be projected to 1.5 billion in 2025. Then, 9.4 million people will potentially die from hypertension and its complications every year [2]. In Southeast Asia, hypertension is a risk factor causing 1.5 million deaths per year [3].

Hypertension is defined as clinical blood pressure at or above 140/90 mmHg [4]. It is believed that managing the hypertension risk could be done by controlling the blood pressure. Controlled systolic blood pressure can reduce the risk of death, cardiovascular disease, stroke, and heart failure. One of the causes of low blood pressure control in hypertensive patients was the lack of adherence to the antihypertensive treatment [5, 6]. Although pharmacological therapy is available, medication adherence is known to be suboptimal because of many factors, such as age, knowledge about hypertension, motivation, and belief in treatment [7]. Other factors include therapeutic regimen [7, 8], the health system that guarantees health financing [9], and treatment information provided by health workers [10].

The importance of monitoring the adherence to antihypertensive drugs can help healthcare staff examine and control patients’ blood pressure. Thus, this study aimed to determine the association of medication adherence with the blood pressure of hypertensive patients. There are two methods of measuring adherence, direct and indirect methods [11, 12]. Direct method is carried out by observing the patients’ condition, such as measuring drug concentration in the patient’s blood samples. In contrast, indirect method for monitoring medication adherence is performed by giving an interview or a questionnaire, calculating the number of drugs, and monitoring drug use [13, 14]. This study was conducted using the pill count method (counting the remaining drug prescribed) to assess medication adherence.

## Methods

### Study design

This research was conducted in five community health centers (locally called *Puskesmas*), including Benowo, Jeruk, Ketabang, Tambak Rejo, and Gayungan. The community health center is the primary health care center covering a sub-district in Surabaya, East Java, Indonesia. Prospective data collection was done in 2 months (April and May 2020). Adherence was assessed by comparing the total number of prescribed antihypertension drugs and the remaining drugs when visiting the community health centers. A direct interview was performed to ask the patients about the number of remaining drugs and the sociodemographic characteristics (age, sex). Type of therapy and comorbidities were recorded from the patient’s medical record at community health centers. Blood pressure was measured by healthcare workers.

### Participants recruitment

The study subjects were purposively selected by following the inclusion and exclusion criteria. The inclusion criteria involved all hypertensive outpatients, including adults and elderly, and currently on antihypertensive treatment. Meanwhile, we excluded patients aged <18 years, pregnant women, patients with mental disorders, and those diagnosed with hypertension for the first time.

### Data analysis

The percentage of patient adherence was calculated by comparing the number of drugs consumed with the number of drugs that had to be consumed then multiplied by 100% [15–17]. In this study, a percentage of pill count <80% and 80–100% were considered non-adherent and adherent, respectively [15]. The blood pressure target in hypertensive patients refers to the hypertension guideline recommendation of Joint National Committee 8 (JNC 8). It further explains that hypertensive patients without comorbid diabetes mellitus and chronic kidney disease at the age of ≥60 years should achieve <150 mmHg systolic blood pressure and <90 mmHg blood pressure diastolic. Meanwhile, for patients aged <60 years, the target of achieving systolic blood pressure is <140 mmHg and systolic blood pressure is <90 mmHg. For hypertensive patients with comorbid diabetes mellitus and/or chronic kidney disease at all ages, the target is systolic blood pressure <140 mmHg and diastolic blood pressure <90 mmHg [18].

### Statistical analysis

This study employed IBM SPSS ver. 20 (IBM Corp., Armonk, NY, USA) for the statistical analysis. A chi-square test was used to determine the association between demographic characteristics with medication adherence and blood pressure. Multivariable logistic regression models were fitted to identify the association of explanatory variables with the outcomes. Variables with a P-value less than 0.20 were considered for inclusion in a multivariable logistic regression analysis in which confounders could be controlled. The necessary assumptions of logistic regression were made by checking Hosmer and Lemeshow’s goodness of fittest statistics. Variables with a P-value less than 0.05 in the multivariable logistic regression analysis were considered as statistically significant determinants. Adjusted odds ratio (AOR) with 95% confidence interval (CI) was calculated to measure the strength of the association between the explanatory variable and the outcome variable.

## Results

A total of 264 patients on antihypertension treatment who participated in this study came from Benowo (40 patients), Jeruk (40 patients), Tambak Rejo (74 patients), Ketabang (40 patients), and Gayungan (70 patients). The majority of the study population was female (73.48%), aged 40–61 years old (51.13%), and educated up to high school (35.98%). Most subjects were prescribed a single antihypertensive agent (91.67%), and the remaining received a combination (8.33%). Regarding comorbidities, more than half of patients had diabetes and dyslipidemia (57.95%). The proportion of adherent and non-adherent patients were 77.65% and 22.35%, respectively. Then, according to the JNC 8 standard, patients were classified as having controlled (108 patients, 40.91%) and uncontrolled blood pressure (156 patients, 59.09%) (Table 1).

**Table 1:** Percentage of baseline and clinical characteristics (*n* = 264)

Variable	Frequency (%)
Age (yr)	
20–40	19 (7.19)
41–60	135 (51.13)
>60	110 (41.68)
Sex	
Female	194 (73.48)
Male	70 (26.52)
Education level	
Elementary school	73 (27.65)
Middle school	37 (14.02)
High school	95 (35.98)
College	59 (22.35)
Comorbidity	
No	154 (58.33)
Comorbid presence	110 (41.67)
Type of therapy (antihypertension drug)	
Monotherapy	242 (91.67)
Combination	22 (8.33)
Medication adherence (pill count method)^a)^	
Adherent	205 (77.65)
Non-adherent	59 (22.35)
Blood pressure level^b)^	
Controlled	108 (40.91)
Uncontrolled	156 (59.09)

^a)^ The percentage of pill count <80% and 80–100% were considered non-adherent and adherent, respectively.

^b)^ Blood level pressure according to JNC 8 guideline: hypertensive patients without comorbid diabetes mellitus and chronic kidney disease at the age of ≥60 years should achieve <150 mmHg systolic blood pressure and <90 mmHg blood pressure diastolic. Meanwhile, for patients aged <60 years, the target of achieving systolic blood pressure is <140 mmHg and systolic blood pressure is <90 mmHg. For hypertensive patients with comorbid diabetes mellitus and/or chronic kidney disease at all ages, the target is systolic blood pressure <140 mmHg and diastolic blood pressure <90 mmHg.

Tables 2 and 3 depicted the association of patients’ characteristics to medication adherence and blood pressure control, respectively (using the chi-square test). The result showed that medication adherence had association with blood pressure control only (*P* = 0.000). Meanwhile, age and patient adherence could influence the blood pressure (*P* = 0.005 and *P* = 0.000, respectively). Then, sex, type of therapy, education level, and the presence of comorbid disease did not associate with adherence nor blood pressure.

**Table 2:** Sociodemographic and clinical characteristics of patients according to their medication adherence status (*n *= 264)

Variable	Adherent (*n* = 205)	Non-adherent (*n* = 59)	*P*-value
Age (yr)			0.552
20–40	14 (73.68)	5 (26.32)	
41–60	102 (75.55)	33 (24.45)	
>60	89 (80.90)	21 (19.10)	
Sex			0.145
Female	155 (79.9)	39 (20.1)	
Male	50 (71.43)	20 (28.57)	
Comorbidity			0.283
No	116 (75.32)	38 (24.68)	
Yes	89 (80.91)	21 (19.09)	
Type of therapy (antihypertension drug)			0.265
Monotherapy	190 (78.51)	52 (21.49)	
Combination	15 (68.18)	7 (31.82)	
Education level			0.425
Elementary school	53 (72.6)	20 (27.4)	
Middle school	30 (81.08)	7 (18.92)	
High school	77 (81.05)	18 (18.95)	
College	45 (76.27)	14 (23.73)	
Blood pressure level			0.000
Controlled	100 (92.59)	8 (7.41)	
Uncontrolled	105 (67.31)	51 (32.69)	

Data are presented as number (%)

**Table 3:** Sociodemographic and characteristic of patients according to their blood pressure control (*n *= 264)

Variable	Controlled (*n* = 108)	Uncontrolled (*n* = 156)	*P*-value
Age (yr)			0.005
20–40	3 (15.79)	16 (84.21)	
41–60	49 (36.30)	86 (63.70)	
>60	56 (50.91)	54 (49.09)	
Sex			0.302
Female	84 (76.36)	110 (23.64)	
Male	24 (34.28)	46 (65.72)	
Comorbidity			0.612
No	62 (40.26)	92 (59.74)	
Yes	46 (41.82)	64 (58.18)	
Type of therapy (antihypertension drug)			
Monotherapy	101 (41.74)	141 (58.26)	0.365
Combination	7 (30.43)	16 (69.57)	
Education level			0.099
Elementary school	26 (36.62)	47 (64.38)	
Middle school	22 (59.46.)	15 (40.54)	
High school	37 (38.95)	58 (61.05)	
College	23 (38.99)	36 (61.02)	
Medication adherence (pill count)			
≥80%	98 (47.80)	107 (52.20)	0.000
<80%	10 (16.95)	49 (83.05)	

Data are presented as number (%)

In line with the previous finding, multivariable logistic regression also showed that patients with uncontrolled blood pressure were associated with poor medication adherence or vice versa (Tables 4 and 5; *P* = 0.000). Moreover, uncontrolled blood pressure was associated with those aged <60 years and educated up to middle school. Then, sex, type of therapy, and the presence of the comorbid disease did not associate with poor adherence or uncontrolled blood pressure (Table 5).

**Table 4:** Multivariable logistic regression results of factors associated with poor medication adherence

Variable	AOR (95% CI)	*P*-value
Age (yr)		
20–40	0.963 (0.289–3.211)	0.951
41–60	1.309 (0.637–2.609)	0.444
>60	Ref.	-
Sex		
Female	0.596 (0.305–1.177)	0.316
Male	Ref.	-
Comorbidity		
No	Ref.	-
Yes	0.674 (0.351–1.294)	0.236
Type of therapy (antihypertension drug)		
Monotherapy	Ref.	-
Combination	1.993 (0.695–5.716)	0.199
Education level		
Elementary school	1.783 (0.464–4.262)	0.193
Midle school	1.759 (0.584–5.298)	0.315
High school	0.894 (0.377–2.121)	0.799
College	Ref.	-
Blood pressure level		
Controlled	Ref.	-
Uncontrolled	6.176 (2.785–14.099)	0.000

The reference category is adherent (percentage pill count ≥ 80%)

AOR, adjusted odds ratio; CI, confidence interval

**Table 5:** Multivariable logistic regression results of factors associated with uncontrolled blood pressure level

Variable	AOR (95% CI)	*P-*value
Age (yr)		
20–40	5.814 (1.519–22.252)	0.010
41–60	2.008 (1.146–3.520)	0.015
>60	Ref.	-
Sex		
Female	0.751 (0.397–1.419)	0.378
Male	Ref.	-
Comorbidity		
No	Ref.	-
Yes	0.933 (0.540–1.612)	0.804
Type of therapy (antihypertension drug)		
Monotherapy	Ref.	-
Combination	1.445 (0.519–4.026)	0.373
Education level		
Elementary school	1.147 (0.589–2.539)	0.669
Middle school	0.387 (0.153–0.974)	0.046
High school	1.158 (0.563–2.382)	0.726
College	Ref.	-
Medication adherence (pill count)		
≥80%	Ref.	-
<80%	6.081 (2.672–13.838)	0.000

The reference category is controlled blood pressure

AOR, adjusted odds ratio; CI, confidence interval

## Discussıon

This study examines the association of medication adherence with blood pressure control status among outpatients in primary health care facilities. It was evident that low adherence was significantly associated with uncontrolled blood pressure. Reciprocally, poor blood pressure control was significantly associated with non-adherence to antihypertension treatment. In line with this finding, several studies reported that highly adherent patients were more likely to have controlled blood pressure than those with lower adherence [19–21]. Although the proportion of adherent patients was high (77.65%), clinical consequences of suboptimal medication adherence are negligible, including uncontrolled blood pressure, accelerating disease progression, and increasing hospital admissions due to cardiovascular complications [22].

In this study, we could not find any significant association between medication adherence and patient characteristics (age, sex, type of therapy, educational level, and the presence of comorbidity) in both statistical analyses. In contrast, ample studies proved many independent predictors related to medication adherence. For instance, Khayyat et al. [21] reported that sex, age, and the presence of comorbid other diseases such as diabetes mellitus affect medication adherence. In another study by Kang et al. [23], it is stated that medication adherence has an association with age and the presence of family members, but not with sex, usage duration, and blood pressure.

Then, the comorbidities accompanying hypertension were not associated with poor medication adherence and uncontrolled blood pressure (Tables 4 and 5). Opposed to this finding, several studies revealed that comorbidities such as diabetes mellitus, heart disease, and dyslipidemia affect patient adherence due to the consumption of more complex medications [24–26].

Patient adherence to their antihypertensive drugs indeed affects blood pressure control. However, other predictors of blood pressure control were found in the present study, such as age and education (Table 5). The patient’s age was inversely associated with uncontrolled blood pressure. The youngest group of patients (20–40 years old) were almost six times more likely to have uncontrolled blood pressure compared to those aged >60 (AOR: 5.809; 95% CI: 1.516–22.264; *P* = 0.010) (Table 5). It may be assumed that uncontrolled blood pressure in these particular age groups correlates with their adherence level percentage. In the oldest patients group, adherence level was 81.82%, whereas in the age group of 41–60 years old was 75.55% and even lower in 20–40 years old with 73.68%, even though the difference was not significant. Furthermore, Choi et al. [24] stated that patients aged >50 years had high adherence to the consumption of antihypertensive drugs. Older patients usually have a caregiver who helps take their medication and maintain a healthy lifestyle, thus control blood pressure eventually [23]. Moreover, older age was associated with better medication adherence due to perceived vulnerability and disease severity [27, 28].

The level of education in this study is known not to affect medication adherence or blood pressure control based on the chi-square test. However, multivariable logistic regression found that patients educated up to middle school had higher blood pressure control than college graduates. Other studies also showed inconsistent results. Ayodapo et al. [29] reported that educational level has no considerable effect on medication adherence, while Adisa et al. [30] showed a statistically significant correlation between medication adherence and education. Generally speaking, patients now have unlimited access to information regarding hypertension and the importance of medication adherence from websites, mobile applications, and health workers or pharmacists.

The success of hypertension therapy is influenced by adherence and other factors, such as lifestyle, physical activity, diet, sleep patterns, body mass index, smoking status, and stress [31]. Those factors were not observed in the present study due to data availability and time limitations. Another limitation involves the measuring adherence method (self-reported pill count). This method is subjective and may be inaccurate because the patient might remove the drugs from the container in anticipation of adjusting the number of the drug according to the medication schedule [7, 11, 17]. Besides, the number of respondents was limited, and the distribution of *Puskesmas* did not represent the whole city. So, it is necessary to add the number of respondents from many more *Puskesmas* for future study.

## Conclusions

We found a significant association between medication adherence and blood pressure control. In addition, age was found to be an independent predictor that affects blood pressure. Thus, pharmacists and other healthcare providers should pay attention to improving medication adherence and maintaining controlled blood pressure, particularly for patients below 40 years old.

## Supplementary information

**Additional file 1.**

**Additional file 2.**

## Data Availability

The datasets used and/or analyzed during the current study are available from the corresponding author on reasonable request.

## Abbreviations

WHO: World Health Organization
SBP: Sistole blood pressure
DBP: Diastole blood pressure
AOR: Adjusted odds ratio
JNC: Joint National Committee
PDC: Proportion of days covered
MEMS: Medication Electronic Monitoring System

## References

[1] Ministry of Health of the Republic of Indonesia. Main Results of Riskesdas 2018. Jakarta. https://kesmas.kemkes.go.id/assets/upload/dir_519d41d8cd98f00/files/Hasil-riskesdas-2018_1274.pdf. Accessed 1 April 2021.

[2] World Health Organization. Fact sheets: Hypertension. 2017. https://www.who.int/news-room/fact-sheets/detail/hypertension. Accessed 13 Feb 2020.

[3] Woodham N, Taneepanichskul S, Somrongthong R, Auamkul N (2018). Medication adherence and associated factors among elderly hypertension patients with uncontrolled blood pressure in rural area, Northeast Thailand. J Health Res.

[4] Kitt J, Fox R, Tucker KL, McManus RJ (2019). New approaches in hypertension management: a review of current and developing technologies and their potential impact on hypertension care. Curr Hypertens Rep.

[5] Burnier M (2014). Managing ‘resistance’: is adherence a target for treatment?. Curr Opin Nephrol Hypertens.

[6] Bochkareva EV, Butina EK, Kim IV, Kontsevaya AV, Drapkina OM, Leon D (2019). Adherence to antihypertensive medication in Russia: a scoping review of studies on levels, determinants and intervention strategies published between 2000 and 2017. Arch Public Health.

[7] Burnier M, Egan BM (2019). Adherence in hypertension. Circ Res.

[8] Danchin N, Neumann A, Tuppin P, De Peretti C, Weill A, Ricordeau P (2011). Impact of free universal medical coverage on medical care and outcomes in low-income patients hospitalized for acute myocardial infarction: an analysis from the French National Health Insurance system. Circ Cardiovasc Qual Outcomes.

[9] Athiyah U, Rahem A, Setiawan CD (2019). The influence of participation of the Social Security Agency (BPJS) Health on therapeutic success in hypertension patients at Community Health Centers. Res J Pharm Technol.

[10] Burnier M (2017). Drug adherence in hypertension. Pharmacol Res.

[11] Ernawati I, Islamiyah WR, Sumarno (2018). How to improve clinical outcome of epileptic seizure control based on medication adherence? A literature review. Open Access Maced J Med Sci.

[12] Basu S, Garg S, Sharma N, Singh MM (2019). Improving the assessment of medication adherence: challenges and considerations with a focus on low-resource settings. Ci Ji Yi Xue Za Zhi.

[13] Vrijens B, Antoniou S, Burnier M, de la Sierra A, Volpe M (2017). Current situation of medication adherence in hypertension. Front Pharmacol.

[14] Ernawati I, Islamiyah WR (2019). Validity and reliability test of MGLS (Morisky, Green, Levine adherence Scale) Questionnaire (Indonesian version) for epilepsy patients. Jurnal Ilmiah Ibnu Sina.

[15] Vik SA, Maxwell CJ, Hogan DB, Patten SB, Johnson JA, Romonko-Slack L (2005). Assessing medication adherence among older persons in community settings. Can J Clin Pharmacol.

[16] Okatch H, Beiter K, Eby J, Chapman J, Marukutira T, Tshume O (2016). Brief report: apparent antiretroviral overadherence by pill count is associated with HIV treatment failure in adolescents. J Acquir Immune Defic Syndr.

[17] Thompson N, Nazir N, Cox LS, Faseru B, Goggin K, Ahluwalia JS (2011). Unannounced telephone pill counts for assessing varenicline adherence in a pilot clinical trial. Patient Prefer Adherence.

[18] James PA, Oparil S, Carter BL, Cushman WC, Dennison-Himmelfarb C, Handler J (2014). 2014 Evidence-based guideline for the management of high blood pressure in adults: report from the panel members appointed to the Eighth Joint National Committee (JNC 8). JAMA.

[19] Haley WE, Gilbert ON, Riley RF, Newman JC, Roumie CL, Whittle J (2016). The association between Self-Reported Medication Adherence scores and systolic blood pressure control: a SPRINT baseline data study. J Am Soc Hypertens.

[20] Butler MJ, Tanner RM, Muntner P, Shimbo D, Bress AP, Shallcross AJ (2017). Adherence to antihypertensive medications and associations with blood pressure among African Americans with hypertension in the Jackson Heart Study. J Am Soc Hypertens.

[21] Khayyat SM, Khayyat SM, Hyat Alhazmi RS, Mohamed MM, Abdul Hadi M (2017). Predictors of medication adherence and blood pressure control among Saudi hypertensive patients attending primary care clinics: a cross-sectional study. PLoS One.

[22] Herttua K, Tabak AG, Martikainen P, Vahtera J, Kivimaki M (2013). Adherence to antihypertensive therapy prior to the first presentation of stroke in hypertensive adults: population-based study. Eur Heart J.

[23] Kang CD, Tsang PP, Li WT, Wang HH, Liu KQ, Griffiths SM (2015). Determinants of medication adherence and blood pressure control among hypertensive patients in Hong Kong: a cross-sectional study. Int J Cardiol.

[24] Choi HY, Oh IJ, Lee JA, Lim J, Kim YS, Jeon TH (2018). Factors affecting adherence to antihypertensive medication. Korean J Fam Med.

[25] Algabbani FM, Algabbani AM (2020). Treatment adherence among patients with hypertension: findings from a cross-sectional study. Clin Hypertens.

[26] Mazzaglia G, Ambrosioni E, Alacqua M, Filippi A, Sessa E, Immordino V (2009). Adherence to antihypertensive medications and cardiovascular morbidity among newly diagnosed hypertensive patients. Circulation.

[27] Kamran A, Sadeghieh Ahari S, Biria M, Malepour A, Heydari H (2014). Determinants of patient’s adherence to hypertension medications: application of health belief model among rural patients. Ann Med Health Sci Res.

[28] Bandi P, Goldmann E, Parikh NS, Farsi P, Boden-Albala B (2017). Age-related differences in antihypertensive medication adherence in hispanics: a cross-sectional community-based survey in New York City, 2011-2012. Prev Chronic Dis.

[29] Ayodapo AO, Elegbede OT, Omosanya OE, Monsudi KF. Patient Education and Medication Adherence among Hypertensives in a Tertiary Hospital, South Western Nigeria. Ethiop J Health Sci. 2020 Mar;30(2):243–50. 10.4314/ejhs.v30i2.12 PMC7060389 32165814

[30] Adisa R, Ilesanmi OA, Fakeye TO (2018). Treatment adherence and blood pressure outcome among hypertensive out-patients in two tertiary hospitals in Sokoto, Northwestern Nigeria. BMC Cardiovasc Disord.

[31] Gupta R, Guptha S (2010). Strategies for initial management of hypertension. Indian J Med Res.

