# Supplementary material for: Association of adherence measured by self-reported pill count with achieved blood pressure level in hypertension patients: a cross-sectional study
Source: Clin Hypertens. 2022 Apr 15;28:12. doi: 10.1186/s40885-022-00195-5 (PMC9011980; doi:10.1186/s40885-022-00195-5)
Supplement: Supplementary file 1 — Additional file 1. [file 40885_2022_195_MOESM1_ESM.pdf]

BioMed Central
The Open Access Publisher

Change of authorship request form

Please read the important information on page 4 before you begin

This form should be used by authors to request any change in authorship. Please fully complete all sections. Use black ink and block capitals and provide each author's full name with the given name first followed by the family name.

Section 1 Please provide the current title of manuscript

Manuscript ID no. CHYP-D-21-00036

Association of Adherence Measured by Self-Reported Pill Count with Achieved Blood Pressure level in Hypertension Patients: Cross-Sectional Study

Section 2 Please provide the current authorship, in the order shown on your manuscript.

	First name(s)	Family name
1st author	IIN	ERNAWATI
2nd author	EZIAH IKA	LUBADA
3rd author	RIA	LUSIYANI
4th author	RAHMAD AJI	PRASETYA
5th author		
6th author		
7th author		

Please use an additional sheet if there are more than 7 authors.

Section 3: Please provide a justification for change. Please use this section to explain your reasons for changing the authorship of your manuscript. Please refer to the journal policy pages for more information about authorship. Please explain why omitted authors were not originally included on the submitted manuscript.

The co-author (Rahmad Aji Prasetya) is added to complete and explain further about the data collection and processing, analysis and interpretation, literature search, writing manuscript and critical reading based on the reviewers' comments.

BioMed Central
The Open Access Publisher

Change of authorship request form

Section 4 Proposed new authorship. Please provide your new authorship list in the order you would like it to appear on the manuscript.

	First name(s)	Family name (this name will appear in full on the final publication and will be searchable on PubMed and similar databases)
1st author	IIN	ERNAWATI
2nd author	EZIAH IKA	LUBADA
3rd author	RIA	LUSIYANI
4th author	RAHMAD AJI	PRASETYA
5th author		
6th author		
7th author		

Please use an additional sheet if there are more than 7 authors.

Section 5 Author contribution, Acknowledgement and competing interests section. Please use this section to provide revised Author Contribution, Acknowledgement and/or Competing Interests sections of your manuscript, ensuring you state what contribution any new authors made and, if appropriate acknowledge any contributors who have been removed as authors. Please ensure these are updated in your manuscript.

New Competing Interests statement:
There is no conflict of interest in this study and publication

New Author Contributions statement:
Data collection and processing, Analysis and interpretation, Literature search, writing manuscript, critical Reviews from new co-authors (Rahmad Aji Prasetya) for complete this manuscript based on reviewers comment.

New Acknowledgement Section:
No new acknowledgement

State 'Not applicable' if there are no new authors.

BioMed Central
The Open Access Publisher

Change of authorship request form

Section 6 **Declaration of agreement. *All* authors, unchanged, new and removed *must* sign this declaration.**

* please delete as appropriate. Delete all of the bold if you were on the original authorship list and are remaining as an author

	First name	Family name		Signature	Affiliated institute	Date
1st author	IIN	ERNAWATI	I agree to the proposed new authorship shown in section 4 /and the **addition/~~removal~~* of my name to the authorship list.**	[illegible]	AKADEMI FARMASI SURABAYA	27/07/2021
2nd author	EZIAH IKA	LUBADA	I agree to the proposed new authorship shown in section 4 /and the **addition/~~removal~~* of my name to the authorship list.**	[illegible]	AKADEMI FARMASI SURABAYA	27/07/2021
3rd author	RIA	LUSIYANI	I agree to the proposed new authorship shown in section 4 /and the **addition/~~removal~~* of my name to the authorship list.**	[illegible]	AKADEMI FARMASI SURABAYA	27/07/2021
4th authors	RAHMAD AJI	PRASETYA	I agree to the proposed new authorship shown in section 4 /and the **addition/~~removal~~* of my name to the authorship list.**	[illegible]	AKADEMI FARMASI SURABAYA	27/07/2021
5th author			I agree to the proposed new authorship shown in section 4 /and the **addition/removal* of my name to the authorship list.**			
6th author			I agree to the proposed new authorship shown in section 4 /and the **addition/removal* of my name to the authorship list.**			
7th author			I agree to the proposed new authorship shown in section 4 /and the **addition/removal* of my name to the authorship list.**			

Please use an additional sheet if there are more than 7 authors. * please delete as appropriate. Delete all of the bold if you were on the original authorship list and are remaining.

BioMed Central
The Open Access Publisher

Change of authorship request form

Important information. Please read.

- Please return this form, fully completed, to the editorial office. We will consider the information you have provided to decide whether to approve the proposed change in authorship. We may choose to contact your institution for more information or undertake a further investigation, if appropriate, before making a final decision.
- Please note, we cannot investigate or mediate any authorship disputes. If you are unable to obtained agreement from all authors (included those who you wish to be removed) you must refer the matter to your institution(s) for investigation. Please inform us if you need to do this.
- If you are not able to return a fully completed form within **14 days** of the date that it was sent to the author requesting the change, we may have to reject your manuscript. We cannot publish manuscripts where authorship has not been agreed by all authors (including those who have been removed).
- Incomplete forms will be rejected.
